# Counterargument to the editor’s letter about the article “Evaluation of functional disability after Chikungunya infection”

**DOI:** 10.1590/0037-8682-0113-2020

**Published:** 2020-04-22

**Authors:** Cristiane Silvia Panato, Eduardo Durans Figueredo, Daniela Bassi, Ilana Mírian Almeida Felipe da Silva, Wellyson da Cunha Araújo Firmo, Adriana Sousa Rêgo, Flor de Maria Araújo Mendonça Silva

**Affiliations:** 1 Universidade CEUMA, Programa de Pós-Graduação em Gestão de Programas e Serviços de Saúde, São Luís, MA, Brasil.; 2 Universidade CEUMA, Departamento de Biomedicina, São Luís, MA, Brasil.

We appreciate the authors’ interest in our article titled, “Evaluation of functional disability after Chikungunya infection”^1^ doi: (https://doi.org/10.1590/0037-8682-0575-2019). We also appreciate the opportunity to respond to their comments. Their critique of our study mainly focused on the following two issues: 1) use of the term functional disability and 2) use of the Roland-Morris questionnaire. We appreciate the authors’ concerns; however, believe there is a rationale to our methods, which we would like to clarify.

According to the International Classification of Functioning, Disability and Health (ICF), functionality and disability are related to health conditions based on the organs’ and system’s functions and body structure and limitations to activities and social participation in the environment where an individual lives. Thus, functionality is used as a positive aspect, whereas disability is related to negative aspects^2^ ^,^ ^3^. The main objective of ICF is to standardize terms used to describe health and related conditions. Therefore, we agree that the term “functional disability” used in our study, does not correspond to the concepts of the ICF and in future scientific research to describe the negative aspects caused by the Chikungunya (CHIK) infection we will use the term disability.

Another critical point in our study was the assessment instrument used. The Roland-Morris Disability Questionnaire was initially developed to measure physical disability in patients with low back pain^4^. However, in 2010, Sardá Júnior, Nicholas, and Pimenta^5^ validated the Roland-Morris Disability Questionnaire for pain in general (QIRM-g), which is the questionnaire used in our study. Furthermore, the authors emphasize that QIRM-g is a valid and reliable measure for the Brazilian population with chronic pain. It is also noteworthy that among the 24 items in QIRM-g, the term pain is used generally, is related to the interference in activities of daily living and biopsychosocial aspects of the individual affected by it, and does not exclusive assess low back pain.

Additionally, we used QIRM-g because of psychometric characteristics consistent between our study and the study by Sardá Júnior, Nicholas, and Pimenta^5^. Another important tool for people with CHIK is the World Health Organization Disability Assessment Schedule (WHODAS 2.0). However, the validation of this instrument for the Brazilian population occurred after completing data collection for our study^6^. Thus, we agree that future studies should consider using the WHODAS 2.0 tool to assess CHIK-related disability.

## References

[1] Panato CS, Figueredo ED, Bassi D, Almeida Felipe IM, Firmo WCA (2019). Evaluation of functional disability after Chikungunya infection. Rev Soc Bras Med Trop.

[2] Farias N, Buchalla CM (2005). A Classificação Internacional de Funcionalidade, Incapacidade e Saúde da Organização Mundial da Saúde: Conceitos, Usos e Perspectivas. Rev. Bras. Epidemiol.

[3] Di Nubila H, Buchalla CM (2008). O papel das Classificações da OMS - CID e CIF nas definições de deficiência e incapacidade. Rev. Bras. Epidemiol.

[4] Nusbaun L, Natour J, Ferraz MB, Goldenberg J (2001). Translation, adaptation and validation of the Roland-Morris questionnaire - Brazil Roland Morris. Braz. J. Med. Biol. Res.

[5] Sardá JJ, Nicholas MK, Pimenta CAM (2010). Validação do Questionário de Incapacidade Roland Morris para Dor em Geral. Rev. Dor.

[6] Sousa AJS, Silva MC, Barreto MCA, Nunes PB, Coutinho BD, Castro SS (2019). Psychometric properties of WHODAS for use in patients with Chikungunya in Brazil. Fisioter. Pesqui.

